# Brassinosteroid Biosynthetic Gene *SlCYP90B3* Alleviates Chilling Injury of Tomato (*Solanum lycopersicum*) Fruits during Cold Storage

**DOI:** 10.3390/antiox11010115

**Published:** 2022-01-05

**Authors:** Songshen Hu, Tonglin Wang, Zhiyong Shao, Fanliang Meng, Hao Chen, Qiaomei Wang, Jirong Zheng, Lihong Liu

**Affiliations:** 1 Department of Horticulture, College of Agriculture and Biotechnology, Hangzhou Academy of Agricultural Sciences, Zhejiang University, Hangzhou 310024, China; husongshen@zju.edu.cn (S.H.); tlwang@zju.edu.cn (T.W.); 2 Key Laboratory of Horticultural Plant Growth and Development, Ministry of Agriculture, Department of Horticulture, Zhejiang University, Hangzhou 310058, China; shaozy@zju.edu.cn (Z.S.); mengfanliang@zju.edu.cn (F.M.); haochen_ddc@zju.edu.cn (H.C.); qmwang@zju.edu.cn (Q.W.)

**Keywords:** tomato fruit, cold storage, *SlCYP90B3*, antioxidant enzymes, membranous lipolytic enzymes, *SlCBF1*

## Abstract

Tomato is susceptible to chilling injury during cold storage. In this study, we found that low temperature promoted the expression of brassinosteroid (BR) biosynthetic genes in tomato fruits. The overexpression of *SlCYP90B3* (*SlCYP90B3-OE*), a key BR biosynthetic gene, alleviated the chilling injury with decreased electrical conductivity and malondialdehyde. In *SlCYP90B3-OE* tomato fruits, the activities of antioxidant enzymes, including ascorbate peroxidase (APX), catalase (CAT), peroxidase (POD), and superoxide dismutase (SOD), were markedly increased, while the activity of membranous lipolytic enzymes, lipoxygenase (LOX), and phospholipase D (PLD), were significantly decreased when compared with the wild-type in response to cold storage. Furthermore, the expression level of the cold-response-system component, *SlCBF1*, was higher in *SlCYP90B3-OE* fruits than in the wild-type fruits. These results indicated that *SlCYP90B3* might be involved in the chilling tolerance of tomato fruits during cold storage, possibly by regulating the antioxidant enzyme system and *SlCBF1* expression.

## 1. Introduction

Cold storage is widely used to extend the shelf-life and maintain the quality of horticultural products during the postharvest period. However, tomato fruits often suffer from chilling injury during low-temperature storage [1]. Chilling injury symptoms include surface lesions, discoloration, increased water loss and decay, and loss of the ability to develop their full color when exposed to warm temperature [2]. With extensive use of cold-chain transportation, there is a reality need to optimize comprehensive strategies to reduce chilling injury symptoms in tomato.

Several technologies have been used to reduce chilling injury, including genetic engineering, modified atmosphere packaging [3], and chemical treatments, such as ethylene [4], gibberellins [5], methyl jasmonate [6], eugenol [7], and oxalic acid [8]. The possible mechanisms for ameliorating chilling injury by such treatments generally involve the regulation of *SlCBF1* gene expression [7], the induction of proline accumulation [9], the stimulation of arginine pathways [6], and the regulation of the antioxidant system [5].

Brassinosteroids (BRs), which are polyhydroxylated steroidal phytohormones, have many important roles in plant vegetative and reproductive growth as well as response to abiotic stresses [10]. Aghdam and Mohammadkhani. reported that exogenous brassinolide (BL) treatment alleviated tomato fruit chilling injury, along with lower malondialdehyde (MDA) accumulation [11]. In addition, the reduction of membranous lipolytic enzyme activities in tomato fruit treated with BL increased the resistance to chilling injury through a reduction in lipid peroxidation and an improvement in cell membrane integrity [11]. In bamboo shoots, exogenous BR treatment alleviated chilling injury by maintaining a higher energy charge and slowing the decrease of the ATP content [12]. In lotus root, the attenuation of chilling injury by BR treatment was caused by lower phenolic accumulation via the inhibition of the activity of phenylalanine-ammonia lyase and polyphenol oxidase and higher ascorbic acid contents via improving the activity of POD, CAT, and APX [13]. In banana fruit, BR treatment could significantly upregulate proteins related to energy biosynthesis, stress response, and cell-wall modification with reduced electrical conductivity and the content of malondialdehyde (MDA), thereby reducing chilling damage [14]. Although the functions of exogenous BRs in response to cold stress have been investigated, the mechanism of endogenous BR biosynthesis in response to cold in tomato fruit remains unclear. The BR synthesis pathway is well-studied. SlCYP90B3 (DWF4) was confirmed to catalyze the early stage of the C22 α-hydroxylation reaction [15]. It has been revealed that *SlCYP90B3* expression is positively related to the BR contents in tomato fruit [16]. In the tomato plant, study has shown that the BR contents of tomato plants are upregulated under cold stress [17]. The role of BR biosynthesis in the cold tolerance of tomato fruit needs further study.

Thus, in the present study, the expression pattern of BR biosynthetic genes in response to cold storage was analyzed. In addition, chilling injury index, MDA content, electrical conductivity, antioxidant enzyme activities, membranous lipolytic enzyme activities, and the *SlCBF1* gene expression of the *SlCYP90B3* overexpression (*SlCYP90B3-OE*) lines and wild-type (WT) were examined to investigate the regulatory role of endogenous BR in the cold tolerance of tomato fruit.

## 2. Materials and Methods

### 2.1. Fruit and Treatment

*SlCYP90B3-OE* (OE-4, OE-5) tomatoes were used as described previously [16]. Tomato fruits (*Solanum lycopersicum* cv. Ailsa Craig) of *SlCYP0B3-OE* (OE-4, OE-5) and WT were harvested in a climate-controlled greenhouse (25 °C day/18 °C night). Mature-green fruit with uniform shape, size, and color and without blemishes or disease were selected. The storage temperature and humidity of tomato fruits were 4 ± 1 °C and 80–90%, respectively. Fruits were split into three groups, WT, *SlCYP0B3-OE* (OE-4), and *SlCYP0B3-OE* (OE-5). Each group contained 180 tomato fruits.

Nine fruits were sampled randomly for gene expression analysis. Fifteen fruits were sampled for electrical conductivity, MDA content, and analysis of antioxidant enzymes. In addition, 20 fruits were subjected to 14 and 28 days of cold storage plus a further 3 days at 20 °C to induce chilling injury symptoms [4]. The mesocarp of fruits was cut into pieces. All samples were stored at −80 °C after being frozen in liquid nitrogen. 

### 2.2. Chilling Injury Index

The chilling injury (CI) index was measured according to the previous report with few modifications [8]. The following four-stage scale reflects the degree of chilling injury of tomato fruits: scale 0, no visible symptoms; scale 1, less than 10% of the surface area of tomato fruit with chilling damage; scale 2, from 10% to 25% surface area of tomato fruit with chilling damage; scale 3, from 25% to 50% surface area of tomato fruit with chilling damage; and scale 4, more than 50% surface area of tomato fruit with chilling damage (Figure S1). Chilling injury index (%) = {Σ [(CI scale) × (number of fruit within the CI scale)]/(total number of fruit) × 4} × 100%.

### 2.3. Malondialdehyde and Electrical Conductivity

MDA content was measured according to the previous report [18]. Frozen tomato powder was homogenized in a 10 mL test tube, and 2 mL 0.6% TBA was added. Then the solution was immersed in boiling water for 20 min, cooled in an ice bath for 20 min, and centrifuged at 10,000× *g* for 15 min. The absorbance at 450 nm, 532 nm, and 600 nm was measured. Electrical conductivity was measured as previously described with slight modification [8]. Five mesocarp discs were obtained from the equatorial region of five fruit, rinsed with distilled water, and wiped dry. Then the mesocarp discs were put into distilled water (30 mL) for 3 h and boiling water for 15 min. The final conductivity (C_1_) was measured after being cooled to room temperature. The initial conductivity (C_0_) and the final conductivity (C_1_) were determined using conductivity meter (DDS-307A, Shanghai, China). Electrical conductivity = C_0_/C_1_ × 100%.

### 2.4. RNA Extraction 

Total RNA extraction was performed according to the previous study [16]. Briefly, frozen tomato powder (0.1 g) was weighed in a 1.5 mL centrifuge tube; 1 mL of RNAiso Plus (Takara, Japan) was added, and it rested for 4 min. Chloroform (200 μL) was added, the mixture was shaken vigorously for 30 s, and it was centrifuged at 4 °C for 15 min at 12,000× *g*. Isopropanol (0.5 mL) was added to the supernatant, gently mixed and allowed to stand for 5 min at room temperature, then centrifuged for 15 min at 12,000× *g*. The supernatant was discarded and 800 μL of 75% ethanol was added. Fifty μL of DEPC H_2_O was added to dissolve the air-dried RNA. 

### 2.5. qRT-PCR Analysis

RNA (1 μg) was reverse-transcripted into cDNA. iCycler (Bio-Rad Inc, Hercules, CA, USA) was used for qRT-PCR analysis. The *SlACTIN7* gene was used as a control for data standardization to correct the quantitative difference in the cDNA, and the 2^−ΔΔCT^ method was generally used for relatixve gene expression analysis [19]. The primer sequences are listed in Table S1.

### 2.6. Determination of Enzyme Activity

The activities of the antioxidant enzymes (APX, CAT, POD, SOD), and membranous lipolytic enzymes (LOX, PLD) released from tomato fruit were detected by a specific ELISA kit (Jiangsu Mei Biao Biological Technology Co., Ltd., Yancheng, China) according to the instructions [20]. Briefly, 1.0 g of frozen tomato sample was homogenized with 9 g of phosphate-buffered saline (PBS, pH 7.2–7.4), and the sample mix was grounded vigorously, then entrifuged at 5000× *g* for 20 min, and the supernatant was carefully collected for the measurement of enzyme activities. All the activities of the antioxidant and membranous lipolytic enzymes are expressed as units per gram protein (U g^−1^ protein).

### 2.7. Statistical Analysis

All experiments used a completely randomized design. SPSS 19.0 software (SPSS Inc., Chicago, IL, USA) was used to analyze the data. The Student’s *t*-test (*p* < 0.05) was used for pairwise comparisons, while the Duncan test (*p* < 0.05) of ANOVA was used for the analysis of multiple comparisons.

## 3. Results and Discussion

### 3.1. BR Biosynthetic Genes Could Be Induced in Response to Cold Storage

**Figure 1:**
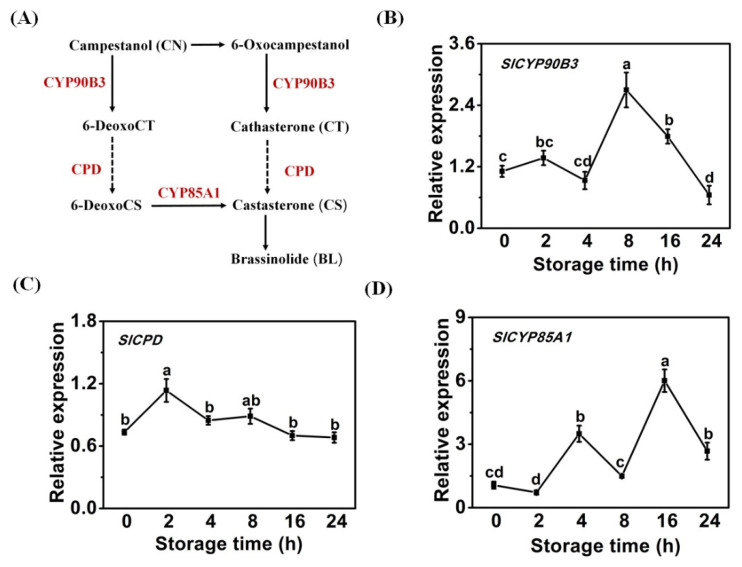
BR biosynthetic genes of tomato fruits were induced in response to cold storage. (**A**) BR biosynthetic pathway in tomato. (**B**) *SlCYP90B3* expression. (**C**) *SlCPD* expression. (**D**) *SlCYP85A1* expression. Each data point is means ± SE (*n* = 3).

Low-temperature storage is an effective and common method used to extend the shelf life of fresh horticultural products. However, a limitation for originally tropical fresh produce is that they are susceptible to chilling injury [1]. Some studies have shown that BR treatment could enhance chilling tolerance in postharvest horticultural products, including tomato [11], bamboo shoot [12], lotus root [13], banana [14], and mango [21]. However, in tomato fruit, the effect of endogenous BR biosynthesis in response to chilling injury remains unknown. The BR contents are upregulated during tomato fruit ripening [16]. To gain more strategies for alleviating or retarding tomato fruit chilling injury, the role of BR in cold stress response was investigated. The pathway of BR biosynthesis in tomato has been well-studied (Figure 1A). SlCYP90B3, SlCPD, and SlCYP85A1 are the specific enzymes that catalyze the synthesis of BRs in tomato. SlCYP90B3 catalyzes the early stage of the C22 α-hydroxylation reaction [15]. CPD catalyzes C-3 oxidation of BR biosynthesis [22]. The tomato *CYP85A1* (*DWARF*) encodes a cytochrome P450, which catalyzes C-6 oxidation [23]. Relative expression levels of these three genes during cold storage were examined (Figure 1B–D). Along with the chilling process, low temperature could result in the enhancement of the *SlCYP90B3* expression up to a peak at 8 h (Figure 1B). *SlCPD* expression reached a peak at 2 h and then declined (Figure 1C), whereas the expression of *SlCYP85A1* reached peaks at 4 h and 16 h (Figure 1D). Thus, it is conceivable that BR may play a specific role in the cold tolerance of tomato fruits.

### 3.2. SlCYP90B3 Overexpression Alleviated Tomato Fruit Chilling Injury

**Figure 2:**
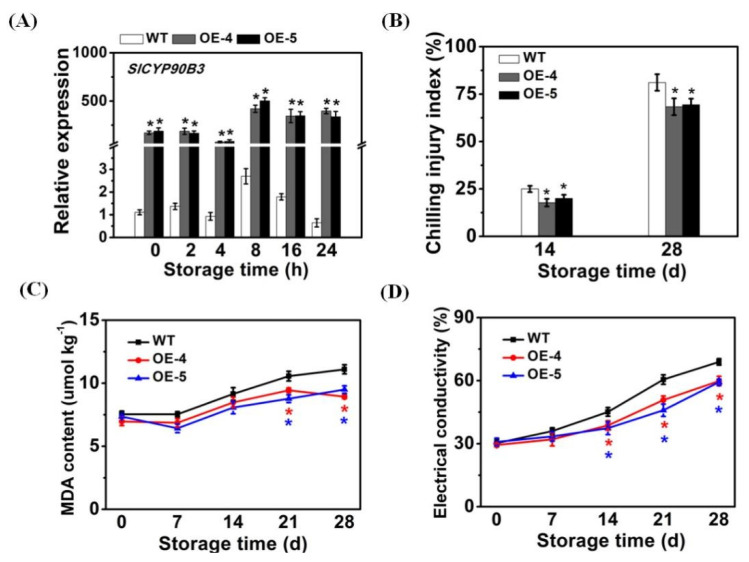
*SlCYP90B3* overexpression alleviated tomato fruit chilling injury. (**A**) *SlCYP90B3* expression in *SlCYP90B3* overexpression and wild-type tomato fruits. (**B**) Chilling injury index. (**C**) MDA content. (**D**) Electrical conductivity. Tomato fruits were stored for 28 days. Each data point is mean ± SE (*n* = 3).

Then, we evaluated the effect of *SlCYP90B3* overexpression on tomato fruit chilling injury to illustrate the mechanism of BR-regulated cold tolerance. The expression level of *SlCYP90B3* maintained upregulation in the transgenic tomato fruit during cold storage (Figure 2A). The chilling injury symptoms that occur in tomato fruit include surface lesions, discoloration, and increased water loss [2,24]. Chilling injury index from WT and *SlCYP90B3-OE* transgenic fruit showed a pattern of increase during the storage periods and the chilling injury symptoms appeared in fruit after 14 days of cold storage plus 3 days at room temperature. The chilling injury index in *SlCYP90B3-OE-4* and *SlCYP90B3-OE-5* were 14.4% and 13.7% lower, respectively, than those of WT at 28th day (Figure 2B). The MDA contents and electrical conductivity were elevated in both WT and *SlCYP90B3-OE* transgenic fruit during cold storage (Figure 2C,D). *SlCYP90B3-OE* fruit had lower MDA levels than WT on day 21 and day 28 during cold storage. MDA content in *SlCYP90B3-OE-4* and *SlCYP90B3-OE-5* on day 28 accumulated to a maximum of 8.93 μmol kg^−1^ and 9.5 μmol kg^−1^, respectively, which were significantly lower than the peak value in WT (Figure 2C). The electrical conductivity increased over the storage time. However, the electrical conductivity from tomato fruit was significantly lower in the *SlCYP90B3-OE* fruit over storage from 14 to 28 days when compared with WT fruit (Figure 2D). The higher expression level of BR biosynthetic gene and decreased chilling injury index, electrical conductivity, and MDA content in the *SlCYP90B3-OE* fruit under low-temperature condition suggested that the overexpression of *SlCYP90B3* increased the resistance of tomato fruits to chilling injury (Figure 2B and Figure S2). Previously, we found that *SlCYP90B3* plays a positive role in regulating tomato fruit ripening, including softening and increases in the contents of soluble sugar, flavor volatiles, carotenoids, and ethylene production. Manipulating *SlCYP90B3* expression in tomato fruits could improve the visual, nutritional, and flavor qualities without yield penalty, improving pre-harvest commodity values [16]. In the present study, the enhanced cold tolerance by *SlCYP90B3* overexpression represents the application value of this gene on tomato post-harvest quality. The regulatory mechanism requires further investigation.

### 3.3. SlCYP90B3 Overexpression Increased APX, CAT, POD, and SOD Activities in Response to Cold Storage

**Figure 3:**
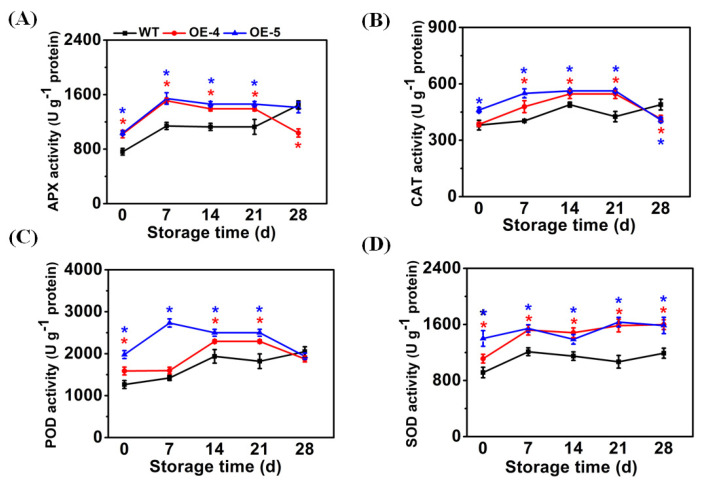
*SlCYP90B3* overexpression increased the antioxidant enzymes activities in tomato fruits during 28 days of cold storage. (**A**) APX, (**B**) CAT, (**C**) POD, and (**D**) SOD. Each data point is mean ± SE (*n* = 3).

Chilling injury symptoms are considered to be caused by ROS accumulation [24]. Antioxidant enzymes, including APX, CAT, POD, and SOD, are part of the well-known oxidative stress defense system to scavenge ROS. The balance between SOD, CAT, APX, and POD activities is critical to cell survival and plays positive role in the regulation of chilling resistance and the maintenance of postharvest fruit quality [25]. Compared with WT fruit, antioxidant enzymes activities, including APX, CAT, POD, and SOD, were significantly higher in *SlCYP90B3-OE* fruit at day 0, indicating that *SlCYP90B3* overexpression increased the basal levels of antioxidant enzymes activities (Figure 3). The activity of APX in WT fruit increased to a maximum of 1447.29 U g^−^^1^ on day 28. APX activity in *SlCYP90B3-OE-4* and *SlCYP90B3-OE-5* increased to a maximum of 1511.02 U g^−1^ and 1542.60 U g^−1^, respectively, on day 7, then declined. APX activity in *SlCYP90B3-OE-4* and *SlCYP90B3-OE-5* were 32.5% and 35.7% higher, respectively, than those in WT on day 7 (Figure 3A). Compared with WT fruit, CAT activity in the *SlCYP90B3-OE* fruit was significantly higher on day 7, day 14, and day 21 (Figure 3B), which reached a maximum of 546.09 U g^−^^1^ and 562.51 U g^−1^ on day 14, respectively. The increases in POD activity in the *SlCYP90B3-OE-4* and *SlCYP90B3-OE-5* fruit compared with that in WT were 18.4% and 29.1% on day 14 and 25.9% and 37.4% on day 21, respectively (Figure 3C). Throughout the whole cold storage period, *SlCYP90B3-OE* fruit showed a significantly higher SOD activity when compared with WT fruit (Figure 3D). Superoxide dismutase (SOD) activity in WT fruit increased to a maximum of 1189.73 U g^−1^ on day 28. SOD activity in *SlCYP90B3-OE-4* increased sharply to the value of 1520.03 U g^−1^ on day 7, then up to a maximum of 1597.9 U g^−1^ on day 28. SOD activity in *SlCYP90B3-OE-5* increased slowly to the value of 1542.92 U g^−1^ on day 7, then kept the activity level with some fluctuation on day 14, day 21, and day 28. The coordination of antioxidant enzymes including SOD, APX, CAT, and POD helps to reduce oxidative damage [26]. SOD is a group of metalloenzymes that dismutate superoxide radicals to molecular oxygen and hydrogen peroxide. The latter product is converted to water by enzymes such as CAT, APX, and POD [27]. After storage for 28 days, among the four antioxidant enzymes, only SOD remained higher activities in *SlCYP90B3-OE-4* and *SlCYP90B3-OE-5* than in WT. The results imply that SOD plays a dominant role and the contents of substrates, including superoxide radicals and hydrogen peroxide, were reduced. In pomegranate fruit, arginine treatment was found to reduce the accumulation of H_2_O_2_ and attenuate chilling injury through increasing the antioxidant enzyme activities [28]. In green bell pepper, brassinolide treatment significantly increased chilling tolerance, and that was accompanied by the promotion of antioxidant enzyme activities [29]. The results showed that the manipulation of *SlCYP90B3* to promote the BR biosynthesis could enhance antioxidant capacity and, consequently, increase chilling tolerance.

### 3.4. SlCYP90B3 Overexpression Suppressed LOX and PLD Activities in Response to Cold Storage

**Figure 4:**
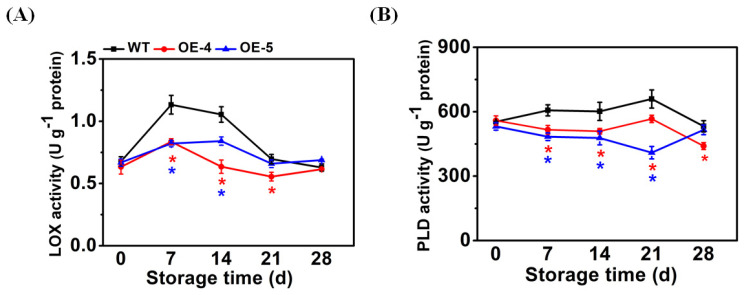
*SlCYP90B3* overexpression suppressed the activities of (**A**) LOX and (**B**) PLD in tomato fruits during 28 days of cold storage. Each data point is mean ± SE (*n* = 3).

When plants are under chilling stress, the cell membrane undergoes a phase transition from a flexible liquid crystalline to a solid gel structure [30], and the content of membrane unsaturated fatty acids will also decrease [31]. PLD and LOX enzymes catalyze the degradation of unsaturated fatty acids. Moreover, LOX and PLD enzymes activities could be activated by low temperature and contribute to maintaining the structure and function of cell membranes [32,33,34]. The enzymatic activities of LOX and PLD were lower in the *SlCYP90B3-OE* fruit when compared with WT fruit. Lipoxygenase (LOX) activity in WT fruit increased to a maximum of 1.13 U g^−1^ on day 7, then declined to 0.63 U g^−1^ on day 28, but the peak values in *SlCYP90B3-OE-4* and *SlCYP90B3-OE-5* were 26.2% and 27.5% lower, respectively, than in WT fruit. During cold storage, *SlCYP90B3-OE* fruit showed lower LOX activity than WT fruit on day 7 and day 14 (Figure 4A). Phospholipase D (PLD) activity in WT fruit increased to a maximum of 659.34 U g^−1^ on day 21, then declined to 532.24 U g^−1^ on day 28. *SlCYP90B3-OE* fruit showed lower PLD activity than WT fruit on day 7, day 14, and day 21. (Figure 4B). In loquat fruit, hot air treatment reduced chilling injury by decreasing the activities of LOX and PLD [35]. Aghdam et al. revealed that, in tomato fruit, reduced chilling injury symptoms by salicylic acid treatment was correlated with the decrease of LOX and PLD activities [36]. In our study, we found that activities of LOX and PLD were lower in *SlCYP90B3-OE* fruit when compared with WT fruit (Figure 4). The inhibited activities of LOX and PLD under chilling stress might be due to the reduced chilling injury in *SlCYP90B3*-overexpressing tomato fruits.

### 3.5. SlCYP90B3 Overexpression Induced the Expression of S lCBF1 during Cold Storage

**Figure 5:**
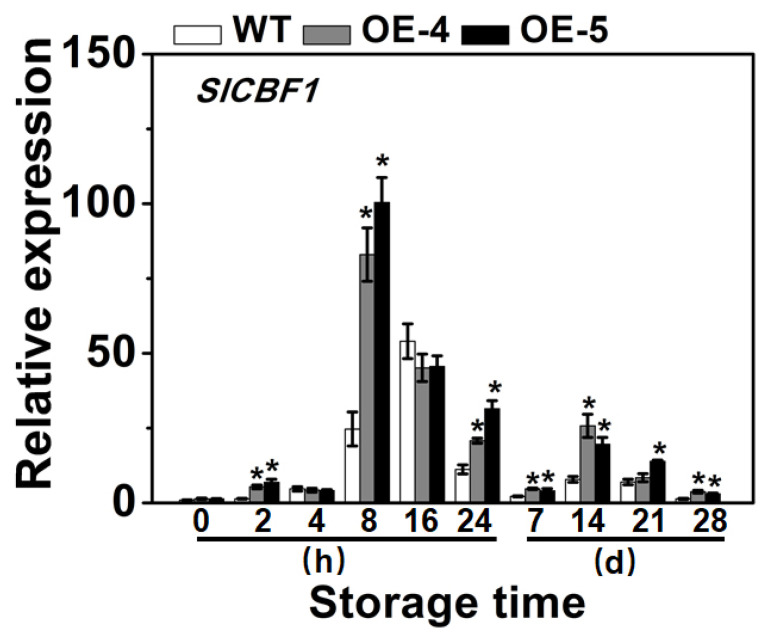
*SlCYP90B3* overexpression induced the expression of *SlCBF1* in tomato fruits during 28 days of cold storage. Each data point is means ± SE (*n* = 3).

C−repeat binding factors (CBFs), upregulated by cold stress, are transcription factors that play a prominent role in the regulation of plant chilling resistance [37]. Three CBF genes (*SlCBF1*, *SlCBF2*, and *SlCBF3*) have been characterized in tomato. Among them, *SlCBF1* is positively correlated with the chilling resistance of tomatoes and negatively correlated with the chilling injury index [4,38]. The tomato *slcbf1* deletion mutant reduced resistance to chilling injury [39]. Several endogenous signal molecules and exogenous treatment could enhance tomato fruit chilling tolerance by regulating *CBF* expression [4,7,40]. To explore the effect of *SlCYP90B3* overexpression on the *SlCBF1* gene expression, quantitative RT-PCR analysis was performed on the tomato fruit subjected to cold storage. *SlCBF1* expression in all samples was the same, rapidly increasing to a peak and then decreasing. Interestingly, the peak expression of *SlCBF1* occurred at 8 h in the *SlCYP90B3*-OE fruit, which was earlier when compared with WT fruit. The expression levels of *SlCBF1* were increased to 3.4 and 4.0 -fold, respectively, at 8 h cold storage in the fruit of *SlCYP90B3-OE-4* and *SlCYP90B3-OE-5* compared with the WT (Figure 5). Moreover, *SlCBF1* gene expression in the *SlCYP90B3-OE* fruit was significantly higher at 2 h, 8 h, 24 h, 7 d, 14 d, and 28 d when compared with WT fruit (Figure 5). The expression level of *CBF1* increased in plants overexpressing brassinosteroid receptor gene *BRI1* and decreased in BR biosynthesis mutant *cpd* [41]. Taken together, the results showed that *SlCYP90B3* overexpression enhanced tomato fruit cold tolerance, which might be conferred by the promotion effect of BRs on the expression of *SlCBF1*.

## 4. Conclusions

**Figure 6:**
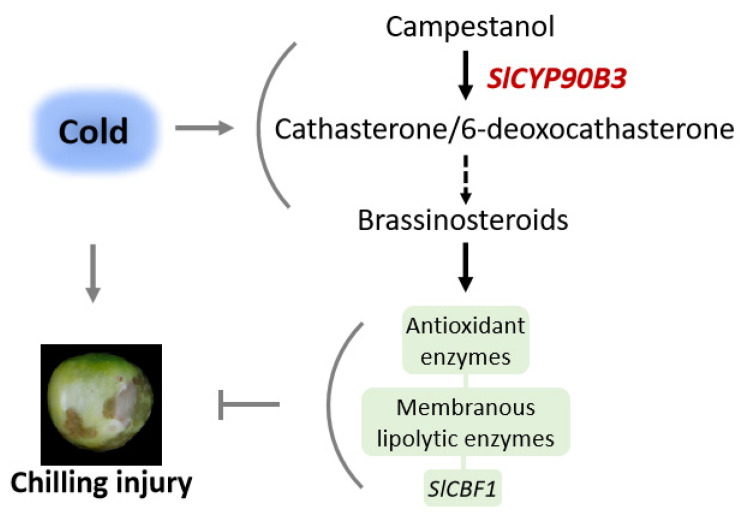
A proposed model of the involvement of the BR biosynthetic gene SlCYP90B3 in the alleviation of chilling injury of tomato fruits during cold storage. Low temperature promoted the expression of BR biosynthetic genes in tomato fruits. The overexpression of the BR biosynthetic gene, *SlCYP90B3*, alleviated chilling injury and enhanced chilling tolerance by promoting the activities of antioxidant enzymes including APX, CAT, POD, and SOD, suppressing the activities of LOX and PLD, as well as upregulating the expression of *SlCBF1*.

In the present investigation, the regulatory role of BR biosynthesis in tomato fruit cold storage was evaluated. The overexpression of the BR biosynthetic gene, *SlCYP90B3*, alleviated chilling injury and enhanced chilling tolerance by promoting the activities of antioxidant enzymes including APX, CAT, POD, and SOD, suppressing the activities of LOX and PLD, as well as upregulating the expression of *SlCBF1* (Figure 6). Overall, these results imply the involvement of BR in the regulation of cold tolerance of tomato fruits. Moreover, *SlCYP90B3* possess the potential to improve the chilling stress adaptation and quality in tomato postharvest storage and could be used for the development of cold-tolerant varieties in the future.

## Data Availability

Data are contained within the article.

## Supplementary Materials

The following supporting information can be downloaded at: https://www.mdpi.com/article/10.3390/antiox11010115/s1, Table S1: Specific primers used for qRT-PCR analysis, Figure S1: Chilled pitting on the surface of postharvest tomato fruits, Figure S2: *SlCYP90B3* overexpression promoted the expression of BR biosynthetic genes during 24 h of cold storage.
