# Supplementary material for: Brassinosteroid Biosynthetic Gene SlCYP90B3 Alleviates Chilling Injury of Tomato (Solanum lycopersicum) Fruits during Cold Storage
Source: Antioxidants (Basel). 2022 Jan 5;11(1):115. doi: 10.3390/antiox11010115 (PMC8773034; doi:10.3390/antiox11010115)
Supplement: Supplementary file 1 [file antioxidants-11-00115-s001.zip › antioxidants-1516343-supplementary.pdf]

Table S1 Specific primers used for qRT-PCR analysis.

**Specific primer sequences used for real-time quantitative PCR analysis.**		
**Target gene**	**Primer sequence (5′ - 3′)**	**gene_name**
SlCYP90B3-F	TGGAGGAGAGGCTTAAGGAA	*Solyc02g085360*
SlCYP90B3-R	GGCCAGCAAAGAGCAAACTC	
SlCPD-F	CTTCTCTCCGAGCTGTTCATCTAG	*Solyc06g051750*
SlCPD-R	GAAGGAAAACAGAGAGTTCCACTC	
SlCYP85A1-F	TCCTGATCCATATTCGTTCAA	*Solyc02g089160*
SlCYP85A1-R	ACCAAGTTCCTTTCCAGGAC	
SlCBF1-F	TTCATCGTCATCGTCGTTTTCT	*Solyc03g026280*
SlCBF1-R	TCCTCTTCCTGATTCCCCTGT	
ACTIN-F	CCTCAGCACATTCCAGCAG	*Solyc03g078400*
ACTIN-R	CCACCAAACTTCTCCATCCC	

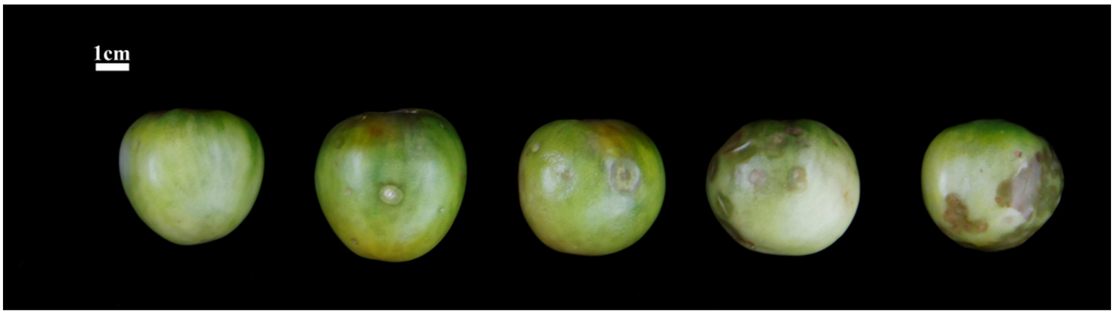

0 (no pitting)　1 (<10%)　2 (10-25%)　3 (25-50%)　4 (>50%)

Figure S1. Chilled pitting on the surface of postharvest tomato fruits.

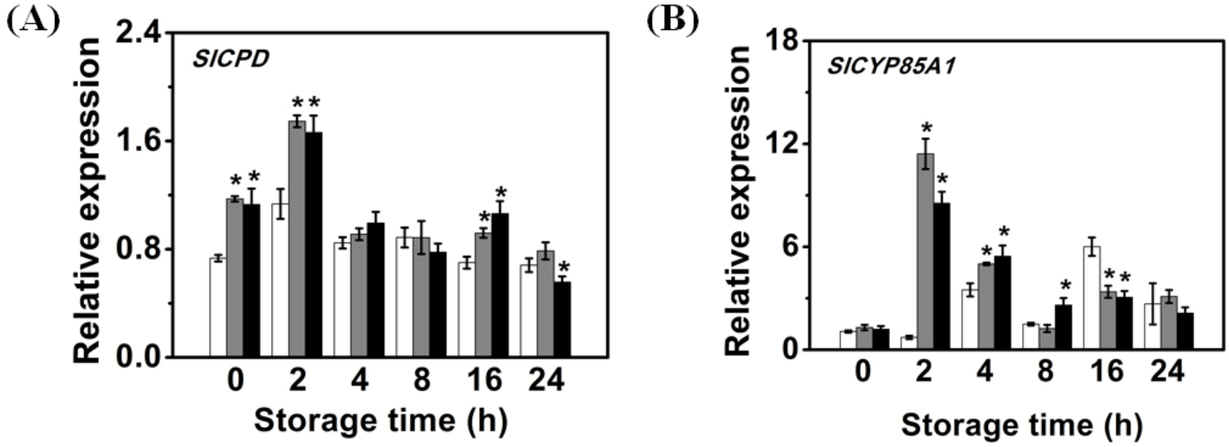

Figure S2. *SlCYP90B3* overexpression promoted the expression of BR biosynthetic genes during 24h of cold storage. (A) Expression levels of *SlCPD*. (B) Expression levels of *SlCYP85A1*. Each data point is means ± SE (n=3).
